# Neurodegeneration in Women

**Published:** 2002

**Authors:** Farida Sohrabji

**Affiliations:** Farida Sohrabji, Ph.D., is an assistant professor in the Department of Human Anatomy and Medical Neurobiology, College of Medicine, the Texas A&M University System Health Science Center, College Station, Texas

Alzheimer’s disease (AD) is a progressive brain disease that results in a dementia accompanied by severe changes in cognitive ability, perception, and mood. Although the precise cause of the disease remains elusive, clusters of specific genetic factors (such as gender and expression of the apolipoprotein E [*ApoE* ] gene) and environmental factors (such as smoking and alcohol use) can alter the risk of AD.

Women appear to be at an increased risk for AD, although women’s longer life spans may contribute to this differential risk. Specifically, the loss of hormones at menopause has emerged as a significant risk factor for AD (Tang et al. 1996; Kawas et al. 1997). With an average life span of 78 years and typical menopausal onset between 48 and 54 years, women spend a significant portion of their lives in an estrogen-deprived state. Because hormonal changes that accompany menopause increase vasomotor dysfunction, hormone replacement therapy has frequently been recommended to symptomatic women. Epidemiological studies of this population, reviewed below, indicate that estrogen replacement at menopause may decrease the risk of neurodegenerative diseases such as AD. This sidebar also examines experimental research designed to determine whether females are more vulnerable than males to alcohol-induced neurocognitive effects.

## Estrogen and Risk for AD

Research shows that estrogen therapy in surgically menopausal women (i.e., women who have experienced induced menopause caused by surgical removal of both ovaries) (Sherwin 1988) and in women receiving therapy to suppress ovarian function (Sherwin and Tulandi 1996) improves cognitive functions such as concentration and attention span. These findings suggest that estrogen may have a protective role in neurodegenerative diseases such as AD.

On the other hand, studies that correlated prior estrogen usage with the incidence of AD have reported mixed findings—some reporting that estrogen is beneficial, others finding that estrogen is detrimental (reviewed in Henderson 1997). As with any retrospective study, data regarding hormone usage were frequently obtained from caregivers and secondary sources, which may not accurately reflect estrogen usage by the patient. More consistent data have been obtained from prospective studies, for which women were followed for several years to determine the effects of estrogen replacement. Results from two recent prospective studies (Tang et al. 1996; Kawas et al. 1997) found that estrogen replacement starting before menopause (i.e., during perimenopause) significantly decreased the risk of AD.

Estrogen therapy does not appear to be beneficial for women with AD, however. Although initial short-term trials with small sample sizes suggested that estrogen replacement was beneficial in female AD patients (Fillit et al. 1986; Honjo et al. 1989), subsequent studies with larger patient populations failed to confirm these findings (Henderson et al. 2000). A recent large-scale, multicenter trial involving female patients with moderate AD reported a modest improvement among patients after 2 months of estrogen replacement but no improvement at 12 months of treatment. Test scores worsened on some measures (Mulnard et al. 2000). These data as well as those from a recent study of cognitively intact postmenopausal women (Drake et al. 2000) underscore the fact that estrogen’s effects on cognition are likely to be complex.

Although the prospective studies and the clinical studies diverge in their conclusions about estrogen’s efficacy, it should be noted that the populations studied in each case are different. Women in the clinical studies were already exhibiting AD symptoms when they entered the study and were therefore likely to have had significant neural damage. Estrogen replacement may therefore be ineffective when hormone-sensitive neurons are lost. Furthermore, the re-introduction of estrogen after a prolonged period of hormone deprivation (approximately 20 years in some studies) may not be an effective replacement strategy.

The contradictory outcomes from the prospective studies and the clinical trials suggest that estrogen use is likely to be prophylactic rather than therapeutic. This hypothesis is supported by experimental evidence in animals which shows that prior treatment with estrogen attenuates forebrain injury caused by inadequate blood supply (i.e., ischemia) (Simpkins et al. 1997; Dubal et al. 1998) and by brain lesions (Sohrabji et al. 2000).

## Gender and Alcohol-Related Neurocognitive Effects

Lifestyle factors such as heavy alcohol consumption may increase the risk for AD among both genders and in females in particular. Although women, on average drink less than men, start drinking later, and consume less alcohol, experimental evidence indicates that females may be more vulnerable to alcohol-induced neurodegeneration than males. Female alcoholics perform worse on tests of recall and psychomotor speed than male alcoholics with similar drinking histories (Acker 1985). Brain imaging studies demonstrate that alcoholic women have specific alterations in the brain that are not found in nonalcoholic women, such as smaller corpus callosums (Hommer et al. 1996) and larger intracranial spaces (Hommer et al. 2001); male alcoholics, however, do not differ from nonalcoholic males on these measures.

Furthermore, on other measures of alcohol-related brain damage, such as ventricle-to-brain-volume ratios (Jacobson 1986) and gray-and-white-matter volumes (Hommer et al. 2001), male and female alcoholics show similar deficits even though females tend to have less severe drinking patterns than males. The lack of gender difference in brain morphology in alcoholics can thus be interpreted to indicate that women are more vulnerable to alcohol toxicity.

Not all studies, however, have shown that females are more vulnerable to alcohol-induced toxicity than males. For example, Pfefferbaum and colleagues (2002) found that female alcoholics were more resistant to age- and alcohol-related changes in some brain structures compared with male alcoholics. Although the reason is unclear, the authors speculate that the difference may be related to estrogen, which has been shown to have beneficial effects on the adult brain. In this study, 75 percent of the alcoholic women were premenopausal and of the remaining women, 62 percent were on postmenopausal estrogen replacement therapy. These data underscore the need for ongoing investigation to determine if women are more vulnerable to alcohol’s neurocognitive effects than men, and to further delineate whether these morphological changes are related to changes in cognition.

Heavy alcohol consumption leads to memory deficits. Research shows that the relationship between alcohol use and dementia appears to follow a J-shaped curve rather than a linear pattern (i.e., one to three drinks per day reduces the risk for dementia, including AD, whereas more than three drinks per day increases the risk for all dementias) (Orgogozo et al. 1997; Cupples et al. 2000; Ruitenberg et al. 2002).

Recent evidence indicates that the nonlinear relationship between alcohol and cognitive deficits may be gender specific (Zuccala et al. 2001). Drinking less than 0.5 liter, or approximately three drinks or less, per day was correlated with improved cognitive ability, compared with those who did not drink, whereas heavy drinking (more than 1.0 liters, or six or more drinks, per day) was negatively correlated with cognitive ability. However, the mid-range dose, defined as between 0.5 and 1.0 liters (between three and six drinks) per day confers cognitive benefits to males but not females. This detrimental effect may be linked to the fact that at comparable doses, females have greater alcohol bioavailability (Baraona et al. 2001). Acetaldehyde, the first metabolite of alcohol, appears earlier in males than females (Zeiner et al. 1983), because of the lower activity levels of the enzyme alcohol dehydrogenase in females (Sato et al. 2001). The resulting increased blood alcohol levels may make females more susceptible to alcohol-related cognitive dementias.

Females’ greater alcohol bioavailability may be associated with alcohol-induced damage to hormone synthesis and metabolism. Alcohol reduces estrogen release during the period preceding ovulation (i.e., proestrus) in the rat (LaPaglia et al. 1997). In humans, alcohol reduces normal release of the form of estrogen known as estradiol in response to human chorionic gonadotropin (hCG) (Gatti et al. 1985). Alcohol also reduces luteinizing hormone–stimulated release of estrogen from ovarian cells grown in culture (Saxena et al. 1990).

The effects of moderate alcohol consumption on estrogen levels may be related to reproductive status. In premenopausal women, moderate alcohol consumption (one drink per day) increases estrogen levels, which is thought to be a possible mechanism by which alcohol may increase the risk of breast cancer. However, in post-menopausal women these data are contradictory, with some studies reporting that alcohol is associated with an increase in estradiol and others reporting no change (summarized in Purohit 1998). Interestingly, alcohol has been found to increase blood estradiol levels in women on estrogen replacement (Ginsburg et al. 1996), suggesting that alcohol may enhance the release of estradiol into blood or slow its degradation, thus ensuring a higher dose of circulating estrogen. Heavy alcohol consumption, however, has not been shown to decrease estrogen levels, although heavy alcohol use prolongs the rodent estrous cycle (Emanuele et al. 2001), specifically the postovulation phase (i.e., diestrus), when blood estrogen levels are low. Prolonging low estrogen stages of the estrous cycle may reduce cumulative hormone stimulation of key neural structures. (See also the article by Emanuele and colleagues in this issue.)

If decreased estrogen exposure potentiates the risk for AD, chronic consumption of alcohol by women may increase the severity or onset of AD. To directly test these hypotheses, longitudinal studies of cognitive changes in heavy and moderate drinkers need to be coupled with regular hormonal profiles and other indicators of estrogen status such as reproductive history and bone density.
